# A multi-organ transcriptome resource for the Burmese Python (*Python molurus bivittatus*)

**DOI:** 10.1186/1756-0500-4-310

**Published:** 2011-08-25

**Authors:** Todd A Castoe, Samuel E Fox, AP Jason de Koning, Alexander W Poole, Juan M Daza, Eric N Smith, Todd C Mockler, Stephen M Secor, David D Pollock

**Affiliations:** 1 Department of Biochemistry and Molecular Genetics, University of Colorado School of Medicine, Aurora, CO 80045 USA; 2 Department of Botany and Plant Pathology and Center for Genome Research and Biocomputing, Oregon State University, Corvallis, OR 97331 USA; 3 Instituto de Biologia, Universidad de Antioquia, Medellin, Colombia; 4 Department of Biology, University of Texas, Arlington, TX 76019 USA; 5 Department of Biological Sciences, University of Alabama, Tuscaloosa, AL 35487 USA

## Abstract

**Background:**

Snakes provide a unique vertebrate system for studying a diversity of extreme adaptations, including those related to development, metabolism, physiology, and venom. Despite their importance as research models, genomic resources for snakes are few. Among snakes, the Burmese python is the premier model for studying extremes of metabolic fluctuation and physiological remodelling. In this species, the consumption of large infrequent meals can induce a 40-fold increase in metabolic rate and more than a doubling in size of some organs. To provide a foundation for research utilizing the python, our aim was to assemble and annotate a transcriptome reference from the heart and liver. To accomplish this aim, we used the 454-FLX sequencing platform to collect sequence data from multiple cDNA libraries.

**Results:**

We collected nearly 1 million 454 sequence reads, and assembled these into 37,245 contigs with a combined length of 13,409,006 bp. To identify known genes, these contigs were compared to chicken and lizard gene sets, and to all Genbank sequences. A total of 13,286 of these contigs were annotated based on similarity to known genes or Genbank sequences. We used gene ontology (GO) assignments to characterize the types of genes in this transcriptome resource. The raw data, transcript contig assembly, and transcript annotations are made available online for use by the broader research community.

**Conclusion:**

These data should facilitate future studies using pythons and snakes in general, helping to further contribute to the utilization of snakes as a model evolutionary and physiological system. This sequence collection represents a major genomic resource for the Burmese python, and the large number of transcript sequences characterized should contribute to future research in this and other snake species.

## Background

A major innovation enabled by next-generation sequencing technologies has been the ability to assemble extensive genomic resources for non-traditional model species. This expanding ability has in turn enabled a renaissance in the use of diverse model species to deliver novel insights not previously possible. Among the emerging model species archetypes are species that demonstrate extreme phenotypes. There is widespread interest in generating necessary genomic resources to facilitate research on these new models of extreme vertebrate phenotypes.

One such group for studying extreme phenotypes are the snakes. Snakes have become increasingly prominent model systems [1], primarily because they represent a vertebrate model system that possesses numerous important extreme adaptations at the morphological and developmental [2-4], physiological and metabolic [5,6], and molecular levels [7-11]. The Burmese python (*Python molurus bivittatus*) in particular has become a focal model system for studying extreme physiological remodelling and metabolic fluctuations that accompany feeding [12-14]. A major problem in studying snakes, however, is that they are highly divergent from other model vertebrate systems that already have genomic resources. The closest vertebrate to snakes with an available complete genome sequence is the *Anolis *lizard (just now being formally published [15]), which last shared a common ancestor with the python ~166 MYA [9,16]. Otherwise, the next closest vertebrates with complete genomes are birds (chicken, finch), which last shared a common ancestor with snakes ~275 MYA [16]. Although some studies have utilized high-throughput sequencing with short reads to study snake transcriptomics, prior to the *Anolis *genome they have been constrained to using bird reference genomes, and have not produced sets of assembled and annotated transcripts [17,18]. Other than the *Anolis *genome, the only existing genomic/transcriptomic resource relevant for studying snakes is a transcriptome data set for the garter snake (*Thamnophis sirtalis*) [19]. Although more closely related than the lizard, this species is also highly evolutionarily distant from the python, as these two species last shared a common ancestor 60-100 MYA [9,16]. Thus, to advance prospects for research utilizing pythons as a model system, a python-specific transcriptome set is needed.

Here, we have assembled a moderate-sized set of transcriptome data from 454 pyrosequencing to create a robust transcriptome reference for future studies utilizing pythons as models for research. We specifically chose to use the more expensive per-base 454 platform for its longer read lengths, which should favor higher assembly accuracy and *de novo *assembly of transcripts. Since our primary goal was to establish a relatively large well-annotated baseline set of snake transcript sequences, we sequenced cDNA libraries generated from multiple sources (heart and liver) and various time points before and after feeding. These sequences were assembled into a combined set of annotated transcript contigs.

## Results and Discussion

### Sequencing and contig assembly

**Figure 1:**
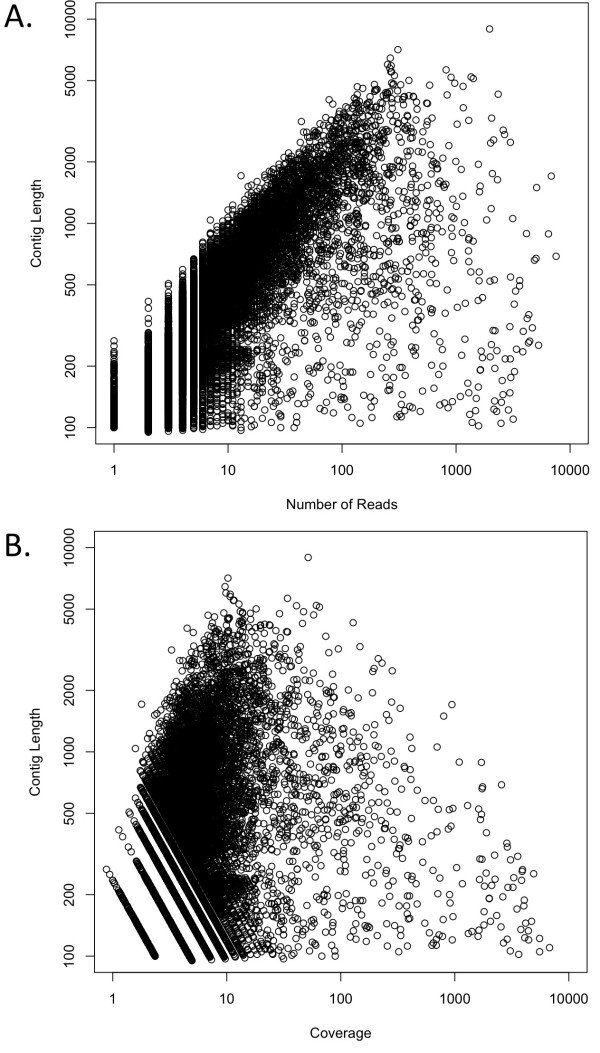
**Contig length versus reads per contig (A) and contig coverage depth (B)**. Results shown on a log scale for all contigs.

**Figure 2:**
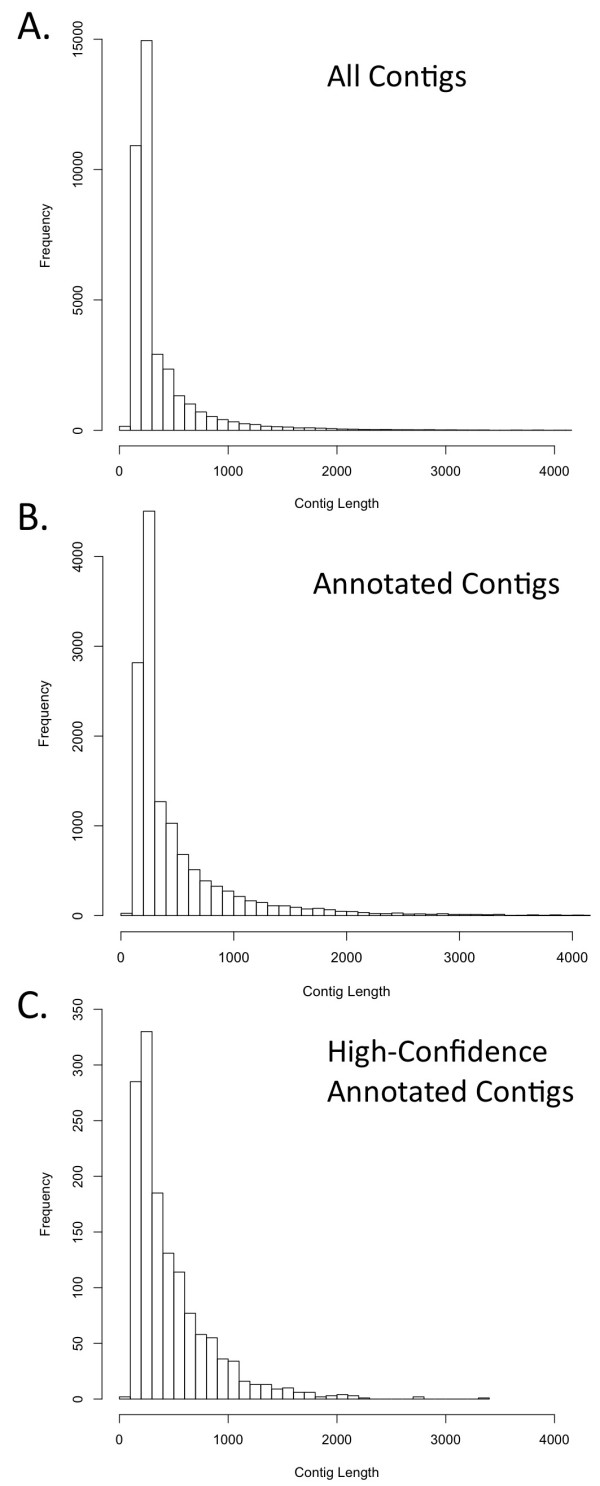
**Contig length distributions for various contig sets**. Frequency distribution of contig lengths for (A) all contigs, (B) all annotated contigs, and (C) all contigs with "high-confidence annotations" in which chicken and lizard one-to-one human orthologous genes *BLAST *results both link to the same human ortholog.

In sum, 983,979 reads totaling >210 megabases (Mbp) were sequenced from python cDNA libraries from heart and liver tissue (Table 1). Combining all cDNA sequences from python heart and liver samples, we assembled 37,245 contigs with a total length of 13,409,006 bp, and with an average GC content of 41.52%. This assembly included 669,607 of our reads, leaving 314,372 singleton reads not incorporated into contigs. Among these contigs, most were sampled by multiple reads, a large number (805) had 100 reads or more (Figure 1). The top 5,000 contigs had lengths greater than 573 bp, and the top 1,000 contigs were longer than 1,420 bp (Figure 1 and 2A).

**Table 1:** Summary of the number of reads and base pairs (bp) collected for tissues and conditions

	Heart Fasted	Heart Fed (24h)	Heart Fed (72h)	Liver	Total
Reads	446,027	215,218	148,230	174,504	983,979
b.p.	104,623,915	38,684,247	27,785,727	38,983,279	210,077,168

Data tabulated includes post quality filtered 454 sequence reads. Raw data are deposited in the NCBI Sequence Read Archive (SRA: SRX018167 and SRX057862)

In the bulk of the data, there is a clear correlation between contig size and read number (Figure 1A), as is expected from random sequencing. The nucleotide-level contig coverage (estimated based on the average read length of ~235 bp) had a mean of 10.5 and a median of 3.5, with 8,084 contigs having > 5 fold average nucleotide coverage. Most contigs are probably close to but not quite full length, since most are covered 2-12 fold with reads at the nucleotide level (Figure 1B).

### Annotation of contigs

We annotated genes based on *BLAST *similarity to known genes in a hierarchical fashion, first based on best *tBLASTx *hits to known Ensembl *Anolis *and chicken genes that are thought to be one-to-one orthologs with human genes. Transcript contigs were also matched to known genes based on *BLASTx *searches against the Genbank non-redundant (nr) protein database (and annotated based on matches), and any remaining genes were annotated based on *megaBLAST *hits to the entire nr nucleotide collection. Of the 37,245 assembled transcript contigs, 13,286 were matched to some known gene through this hierarchical process, and were thus annotated based on similarity to known genes. Thus, we were able to assign some annotation to 35.7% of all contigs. Compared to the length distribution of all contigs (Figure 2A), the distribution of contig lengths for those with any annotation shows a notable enrichment for the annotation of longer contigs (>1,000 bp; Figure 2B).

Among the contigs that were annotated, 3,822 had a best *BLAST *match to known chicken genes that are one-to-one human orthologs, and 4,302 hit known *Anolis *lizard one-to-one human orthologs. Ensembl gene IDs were assigned to transcript contigs based on hits with chicken and *Anolis *genes, and human orthologs were assigned to each contig based on the Ensembl orthologous gene relationship estimates. We considered the annotation of our contigs to be "high confidence annotations" when Ensembl IDs from *Anolis *and chicken *BLAST *hits both linked back to the same human ortholog; 3,046 of our contigs fell into this class (Figure 2C).

**Figure 3:**
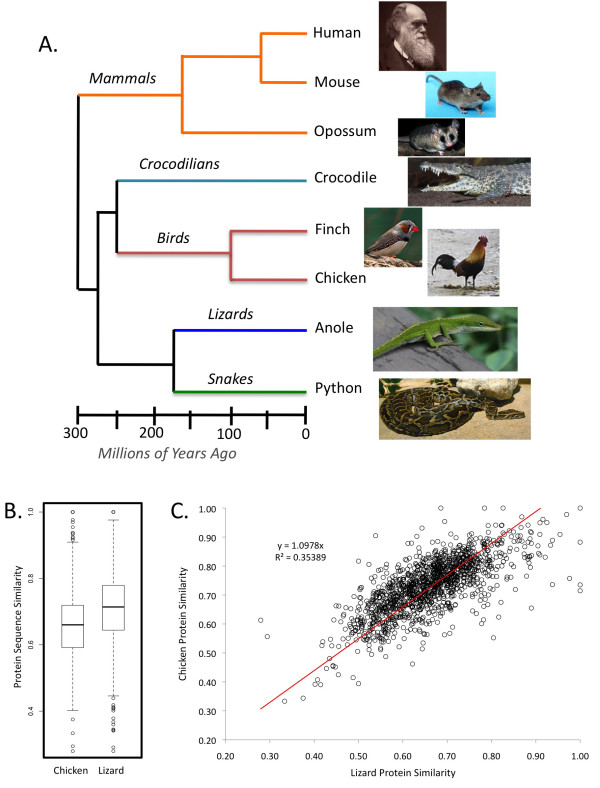
**Comparison of protein divergence between the python, chicken and lizard, and the evolutionary context of the python**. Comparisons of the distribution (A), and linear relationship (B) of protein sequence similarity of the python-chicken versus python-lizard presumed orthologous protein pairs. Only 'high confidence annotated contigs', in which the lizard-snake and chicken-snake matches were to Ensembl orthologous genes, were used for comparisons. Evolutionary context for snakes in relation to major amniote lineages (C); images from Wikimedia Commons.

For contigs with high-confidence annotations, we compared the protein sequence divergence between our python contigs and the lizard and chicken matches. It is estimated that the python and the *Anolis *lizard last shared a common ancestor ~166 MYA [9,16], whereas the chicken and python last shared an ancestor ~275 MY [16] (Figure 3A). Thus, as expected, the protein sequence divergence between the lizard and python proteins (mean = 0.73) is notably less than that between chicken and python proteins (mean = 0.66), although the variation in divergence is quite large (Figure 3B-C). There also is a fairly consistent linear relationship (R^2 ^= 0.35389; Figure 3C) between the python-lizard and python-chicken protein divergence. This indicates that, overall, the protein sequence divergence between python and lizard *BLAST *matches tends to be proportional to that between the corresponding python and chicken *BLAST *matches. This correlation fits the expectation that the chicken and lizard *BLAST *matches tend to be orthologs of one another, wheras poor correlations might indicate *BLAST *matches to lizard and chicken paralogs.

### Gene ontology (GO) analysis

**Figure 4:**
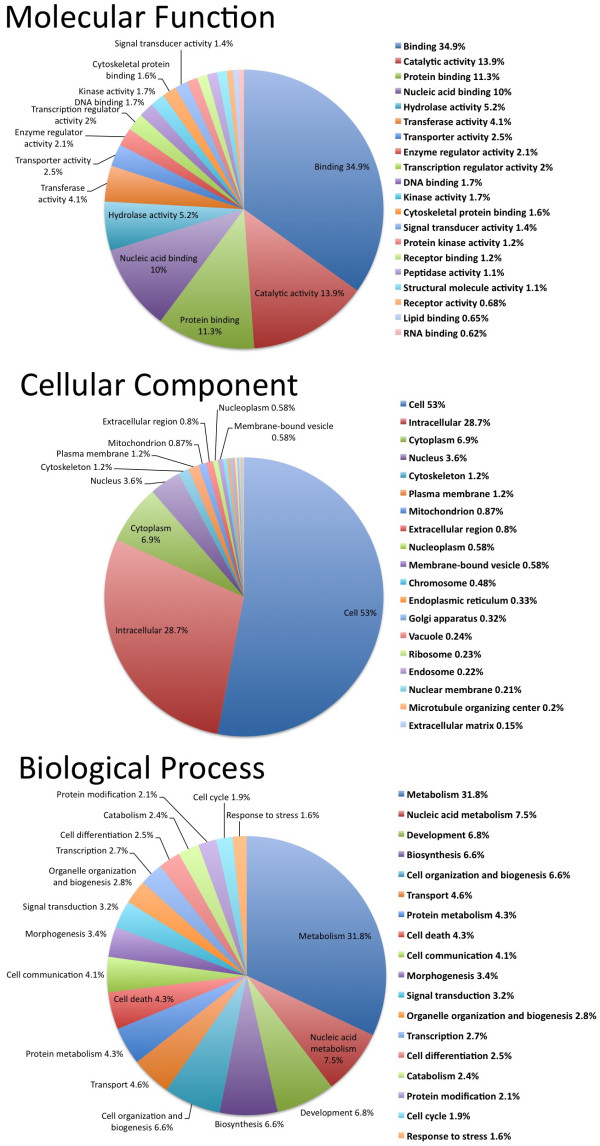
**Gene ontology (GO) categories of the transcriptome set**. Second-level GO annotations are shown based on hits to the Genbank nr database.

For the purpose of GO annotation, we were able to associate GO terms to 12,370 python contigs that were *BLASTx*-matched with known proteins in the NCBI nr database. The frequencies of second-level GO term annotations for our set of 12,370 python matched contigs are shown in Figure 4. Pythons are important models for studying physiological and metabolic remodelling. It is therefore notable that our set of annotated genes includes high frequencies of genes with Biological Process GO annotations that include metabolism, development, cell organization, and morphogenesis that are all likely categories of genes likely to be important for later functional studies of adaptations in pythons (Figure 4).

### Data deposition and accessibility

Raw data from heart cDNA libraries is accessioned in the NCBI Sequence Read Archive (SRA: SRX018167). A minority of the data analysed here, from liver cDNA, were published previously, although not previously assembled [20], and related raw data is accessioned in the SRA (SRA: SRX057862). The set of assembled transcript contigs from this study, together with an extensive table with coordinated information and annotation for contigs, are available online via the journals website (as Additional File 1 and Additional File 2, respectively); these files are also available at http://www.snakegenomics.org/SnakeGenomics/Processed_Data.html.

## Conclusions

Our ultimate goal is to use the python, and other snake species, as models for studying extreme adaptation at various biological levels, from the extreme evolution of proteins [8,10,11] to the extreme systems biology of physiological redesign accompanying feeding [12,21]. We therefore consider it a necessary first step to establish baseline resources, such as this transcriptome set. Here, we chose to use the relatively long sequences available from the 454 platform to conduct *de novo *assembly of transcripts for the python because having such longer sequences is expected to generally favor longer and more accurate transcript assemblies. Additionally, having longer transcript reconstructions is also expected to lead to greater success in identification of orthologous genes in other more well-studies model species, particularly in the case of the python, which is more than ~160 MY diverged from the next closest reference genome of the *Anolis *lizard (Figure 3C).

The results of our *de novo *assembly did indeed produce a relatively large number of long reconstructed transcripts, with nearly 2,000 contigs greater than 1 kb in length. Contrary to expectation, however, we had relatively low success in matching these contigs to known vertebrate genes, with ~35% of contigs matching known genes. Similarly, in a recent analysis of 454-based transcriptome data from diverse tissues for the garter snake, only 34% of transcript contigs were matched to known genes [19]. These numbers for snakes are relatively low compared with percentages of gene identification from other recent transcriptome projects. For example, a recent study on the heart transcriptome of the vole was able to identify ~43% of transcripts based on homology with known mouse transcripts [22].

One obvious explanation for the difficulty in identifying transcripts to known genes for snakes is the relatively low numbers of known genes deposited in NCBI for snakes and reptiles in general. For example, of the ~2.35 million vertebrate proteins on NCBI, 1.61 million are from mammals, compared to ~195,000 for birds, ~90,000 for squamate reptiles (lizards and snakes), and ~24,000 for snakes. Furthermore, because a large proportion of proteins deposited from reptiles are from phylogenetic studies (with one gene sequenced from many species), the diversity of proteins represented is even lower than might be expected from the above numbers. This paucity of genetic information for reptiles highlights the importance for deposition of data from studies like this one, and further argues for the need for additional data to complement our knowledge of amniote genetic diversity.

There are ongoing initiatives to sequence the genomes of the Burmese python [20], as well as the garter snake [1], which should collectively contribute substantial information on reptilian and snake genomics helping to fill a void in our current knowledge of the genomics of amniotes. The genome project for the python will include the addition of more transcriptome data from diverse tissues, and the transcriptome set here will be combined with future data for annotating the python genome [20], and serve as a valuable reference for thousands of annotated python genes in the meantime.

## Methods

### RNA isolation and cDNA library creation

Tissues were procured from a total of 4 animals (one sample per tissue, each tissue from a distinct animal) obtained from commercial pet trade breeders under approved animal care protocols, and stored in RNAlater or snap-frozen in liquid nitrogen prior to RNA extraction. Prior to tissue extraction, two animals were fed and then euthanized either 1 day or 3 days after feeding [13], following existing IACUC protocols in place at the University of Texas Arlington and The University of Colorado.

Total RNA was extracted using Trizol Reagent (Invitrogen), following the manufacturer's protocol. Extracted RNA was enriched for mature mRNA transcripts using three successive rounds of purification with Oligo dT^25 ^beads (PureBiotech), precipitated using linearized acrylamide (Ambion) sodium acetate, and ethanol, and analyzed using a BioAnalyzer pico-RNA chip (Agilent).

The mRNA was reverse transcribed with random heptamers and modified oligonucleotide-dT primers (5'-/Phos/NNNNNNN-3' and 5'-/Phos/TTTTTVN-3') in a 2:1 ratio, using the SuperScript III reverse transcriptase kit (Invitrogen). The remaining RNA was destroyed using RNAse A and RNAse H, and the sample was purified using RNA Clean beads (Ambion). Two pairs of double-stranded adapter oligonucleotides with single-stranded overhang were directionally ligated onto the previously synthesized first strand using T4 DNA Ligase (Invitrogen). Adapter oligonucleotide sequences were: Adapter-A (5-prime adapter), oligo A-prime 5'-NNNNNNCTGATGGCGCGAGGGAGG-dideoxyC-3', and oligo A 5'-GCCTCCCTCGCGCCATGAG-3'; and Adapter-B (3-prime adapter) oligo B 5'-biotin-GCCTTGCCAGCCCGCTCAGNNNNNN-phosphate-3', and oligo B-prime 5'-phosphate-CTGAGCGGGCTGCAAGG-dideoxyC-3'.

Following adapter ligation, ligation products were purified using RNA Clean beads three successive times, and then with streptavidin beads (PureBiotech). Samples were then melted from the streptavidin beads using 0.1M NaOH and precipitated (as above). Completed libraries were then quantified and checked for appropriate size distribution using the DNA-nano chip on a BioAnalyzer (Agilent). Where necessary, libraries were PCR amplified using Platinum Taq polymerase (Invitrogen) using a minimal number of amplification cycles (less than 25 cycles).

### 454-sequencing of cDNA libraries

All cDNA libraries were sequenced using the 454 GS FLX sequencer using the LR70 sequencing kit and 70 × 75 mm PicoTiterPlate (Roche). Emulsion PCR kits II and III (Roche) were used for sequencing cDNA libraries to obtain sequence from both ends of transcripts, because cDNA libraries were directional (with kit II sequencing from the 5' end, and kit III sequencing from the 3' end).

### Assembly of cDNA contigs, and identification of orthologous genes

All of our python cDNA data were assembled into contigs using the Newbler *de novo *assembler algorithm of the *gsassembler *(Roche 454). Contig coverage was estimated by multiplying the number of reads per contig by the average read length divided by contig length. All contigs were compared to the set of *Anolis *(lizard) and chicken Ensembl protein-coding genes that are estimated by Ensembl Compara to be one-to-one orthologs with Human genes using *BLASTx*. When contigs had hits to both chicken and *Anolis *one-to-one orthologs, Ensembl IDs were used to link back to the predicted human ortholog using Ensembl Compara's one-to-one ortholog predictions. If both chicken and *Anolis *hits liked to the same human gene, these were considered 'high-confidence annotated contigs'. Contigs were also compared to the complete NCBI nr database first using *BLASTx *against all proteins (at an *E-value *threshold 10^**-5**^). If contigs had no hits to nr proteins, they were compared at the nucleotide level to all nr sequences *megaBLAST*. We preferentially annotated contigs (with best *BLAST *hits) based first on similarity to *Anolis *and chicken one-to-one orthologs, then based on nr proteins, and finally on nucleotide comparisons where available.

For gene ontology analysis, results of the NCBI nr protein database *BLASTx *search were used to connect python transcript contigs with known gene ontology annotations. Gene ontology annotations were identified using the Blast2GO bioinformatics suite based upon the *BLASTx *output [23]. For the purpose of annotating and displaying GO annotations, we used GO-slims, which depicts second level GO terms that are most conducive to graphical interpretation.

## Competing interests

The authors declare that they have no competing interests.

## Authors' contributions

TC and DP conceived of the study and wrote the paper; TC, JdeK, and SF conducted analyses; TC, AP, JD, and SF conducted laboratory work. All authors participated in editing the manuscript, and approved the final manuscript.

## Links

**Consortium for Snake Genomics website and data clearinghouse **[http://www.snakegenomics.org]
